# Evaluation of immunohistochemical expression of stem cell markers (NANOG and CD133) in normal, hyperplastic, and malignant endometrium

**DOI:** 10.25122/jml-2021-0206

**Published:** 2022-01

**Authors:** Methaq Al-Kaabi, Khalida Noel, Abdal-jabbar Al-Rubai

**Affiliations:** 1. Pathology and Forensic Medicine Department, College of Medicine, Mustansiriyah University, Baghdad, Iraq; 2. Anatomy, Histology and Embryology Department, College of Medicine, Mustansiriyah University, Baghdad, Iraq

**Keywords:** cancer stem cell markers, CD 133, endometrial carcinoma, endometrial hyperplasia, immunohistochemical, NANOG, normal endometrium

## Abstract

Cancer stem cells (CSC) are a potential cause for recurrence, metastasis, and resistance of tumors to different therapeutic modalities like hormonal radiotherapy and chemotherapy. We investigated two CSC markers (NANOG and CD 133) in normal, hyperplastic endometrium and endometrial carcinoma. A total of 93 formalin-fixed paraffin-embedded tissue blocks were used for immunohistochemical expression of NANOG and CD133 markers. NANOG expression was detected in 88.37% of endometrial carcinoma cases compared to 15% of the normal proliferative endometrium and 60% of hyperplasia cases. In endometrial carcinoma, high NANOG expression was significantly correlated with high grade, deep myometrial invasion, lymph node metastasis, and high stage with p-values (0.009, 0.005, 0.014, and 0.003, respectively). CD133 was positive in 76.74% of endometrial carcinoma cases, and it showed a significant correlation with deep myometrial invasion, positive lymph node, positive lymphovascular invasion, and high stage (p-values 0.003, 0.001, 0.003, and 0.013, respectively). Normal endometrium showed less expression of CD133 (only 5%) than hyperplasia and endometrial carcinoma with a statistically highly significant difference (p less than 0.0001). Hyperplastic cases with atypia expressed higher CD133 than those without atypia (6 out of 12 versus 3 out of 18). However, this difference was not statistically significant (p-value 0.111). The cancer stem cell markers NANOG and CD 133 are expressed in a high percentage in endometrial carcinoma compared to normal and hyperplasia and their expression is positively correlated with the aggressive behavior of the tumor. High expression of these two markers in apparently normal tissue around the tumor and in hyperplastic conditions with atypia suggests the possibility to use NANOG and CD133 expression as a diagnostic marker distinguishing dysplasia from reactive atypia. Therefore, inhibition of these markers can be a promising method to stop the progression of early cancers.

## Introduction

Endometrial carcinoma is the most frequent malignancy of the female genital tract in developed western countries [1]. Worldwide, it is the second leading cause of gynecological malignancy [2]. Previous studies suggested the presence of tumor stem-like cells to influence the major processes of tumor progression [3]. These cells can induce and self-renew the tumor and express several genes with pluripotent features [4]. Therefore, these cells were named cancer stem cells identified in many different organ cancers [5] and regarded as a potential cause for recurrence, metastasis, and resistance to different therapeutic modalities like hormonal, radiotherapy, and chemotherapy [4]. Several markers have been identified in cancer stem cells like CD133, CD 166, CD44, CD40, and NANOG [6].

NANOG is an important stem cell transcription factor that participates in normal cell development and tumorigenesis [7]. NANOG regulates embryonic and fetal development and has a crucial role in the preimplantation development phase, with a progressive decrease during embryonic stem cell differentiation. After birth, a limited number of human tissues show a low level of expression in some cells in organs like the testis, ovary, and uterine glands, but most of the tissue is undetectable [8]. Re-expression of NANOG has been detected during carcinogenesis. Many studies identified that NANOG expression is already present in precancerous lesions, with rising levels in high-grade dysplasia. Therefore, it can be used as a diagnostic marker, distinguishing between true dysplasia and reactive lesions [9]. NANOG enables cancer cells to acquire stem-cell-like properties like self-renewal and immortality, leading to growth expansion, tumor maintenance, metastasis formation, and tumor relapse. Cancer showing high NANOG expression is usually associated with high grade, advanced stage, worse overall survival, and resistance to treatment [5].

CD133 is a 97 kDa pentaspan transmembrane glycoprotein. Its function in normal tissue and its role in carcinogenesis remains elusive. Its localization in microvilli and membrane protrusion suggests its role in membrane organization. Subcellular localization of CD133 allows it to connect with lipid rafts involved in the signaling cascade [10]. Studies on both normal cells and cancer stem cells demonstrated that CD133 expression is dependent on the cell cycle [11]. Recently, CD133 has been used as a marker for stem cell identification in several tissues like haematopoetic, brain, and prostate. It has been suggested that CD133 can be used to identify cancer stem cells in variable solid tumors like tumors of the prostate, brain, ovary, colon, liver, and ovary. CSCs with high expression of CD133 show high ability in self-renewing and have a high potential to proliferate to make tumors histologically similar to solid parent tumors after transplanting them in immune-deficient mice [10]. This study aimed (1) to investigate the immunohistochemical expression of cancer stem cell markers NANOG and CD133 in endometrial hyperplasia and endometrial carcinoma and their correlation with different clinicopathological parameters and (2) to compare the expression of these markers in normal endometrial tissue and apparently normal tissue around the tumor.

## Material and Methods

This retrospective study was conducted on tissue samples from 93 female patients admitted to the obstetrics and gynecology department who have undergone hysterectomy to treat endometrial cancer, leiomyoma treatment, and dilation and curettage (D&C) to treat abnormal uterine bleeding.

### Samples

A total of 93 archival formalin-fixed paraffin-embedded tissue blocks were retrieved from AL-Yarmouk hospital from January 2018 until June 2020. The clinical information was collected from the available records. All hematoxylin and eosin-stained slides were reviewed to confirm the initial diagnosis and determine the tumor type, grade, depth of myometrial invasion, and lymphovascular invasion (LVI). All cases showed endometrioid histology. The control group consisted of 20 normal proliferative phase endometrium tissue samples collected from patients who underwent hysterectomy for leiomyoma. The tissue specimens were divided into three groups. The normal endometrial group includes 20 patients with an average age of 25–47 years. The second group of patients with endometrial hyperplasia included 30 patients with an average age of 30–52 years diagnosed with abnormal uterine bleeding. 18 samples showed simple hyperplasia without atypia, while 12 cases demonstrated complex hyperplasia with atypia.

The third group of patients, called endometrial cancer, included 43 patients diagnosed with endometrial cancer, average age 44–57 years. 23 were grade I, 13 were grade II, and grade III was diagnosed in 7 samples. The mass size was less than or equal to 4 in 25 cases. 18 cases were more than 4 cm. Regarding the depth of myometrial invasion, 23 cases were superficial (less than 50%). The deep invasion (more than or equal to 50%) was found in 20 cases.

### Immunohistochemistry

For each case, 3 consecutive sections were obtained with a thickness of 4 μm. One slide was stained with H&E to check and confirm the diagnosis, while the other 2 sections were placed on a positively charged glass slide and stained according to standard staining protocol. Primary anti-CD133 antibody (Abnova, Entrez GeneID 8842, Code PAB12663, Rabbit antihuman polyclonal antibody), and primary anti-NANOG antibody (Abnova clone 60CT77.1.1 Catalog no. MAB12279, mouse antihuman monoclonal antibody). The dilution was 1:200 for CD133 and 1:50 for NANOG using antibody diluent solution (Abcam^®^ USA, code ab64211). Then secondary detection kits for CD133 (Abcam^®^ USA, code ab64261 rabbit-specific HRP/DAB) and NANOG (Abcam^®^ USA, code ab80436 mouse-specific HRP/DAB) were used based on labeled streptavidin-biotin technique.

### Scoring of immunohistochemical staining

Anti-CD133 expression depicted a membranous and/or cytoplasmic brown staining, while anti-NANOG expression showed a distinct nuclear brown staining. Marker immunostaining was scored using the extent of staining (proportion or percentage of stained cells) and staining intensity.

CD133 scoring: We calculated the extent of positive stained epithelial cells and classified them into 4-point scale as the following: No staining=0%, 1=1–10%, 2=11–25%, 3=26–50% and 4=51–100%. Staining intensity was categorized into three groups: weak (+1), moderate (+2), and strong (+3). The final score was calculated by multiplying the extent score by staining intensity. The combined immunohistochemical score ranged from 0 to 12. The cut-off point of 10 was used to segregate cases with weak to moderate expression from cases with strong immune expression.

The final categorization, according to the above, divided cases into 3 groups: 0 = (Absent), Immunohistochemical stain (IHS) ≤10 and IHS >10 (Strong) [12].

Regarding NANOG that show a predominant nuclear expression, the staining intensity for positive cells was calculated and classified into 4 scores: 0 – (No Staining), 1 – (Weak Staining), 2 – (Moderate Staining), 3 – (Strong Staining). The percentage of positive tumor cells was also scored as: 0 = (None of the tumor cells), 1 = (1–50% of positive tumor cells), 2 = (51–100% of positive tumor cells). Then the total score with the formula (percentage of positive tumor cells X staining intensity) was obtained; the total score ranged from 0–6. The results were classified into low (0–3) and high (4–6) [7].

### Statistical analysis

Data analysis was performed using SPSS (version 24). Qualitative variables were analyzed using percentage, mean, and range. Qualitative variables were statistically analyzed using the Pearson Chi-square test and Fisher exact test to obtain the significance of the relationship between clinicopathological characteristics and NANOG and CD133 expression. A p-value of less than 0.05 was considered statistically significant.

## Results

**Figure 1.**
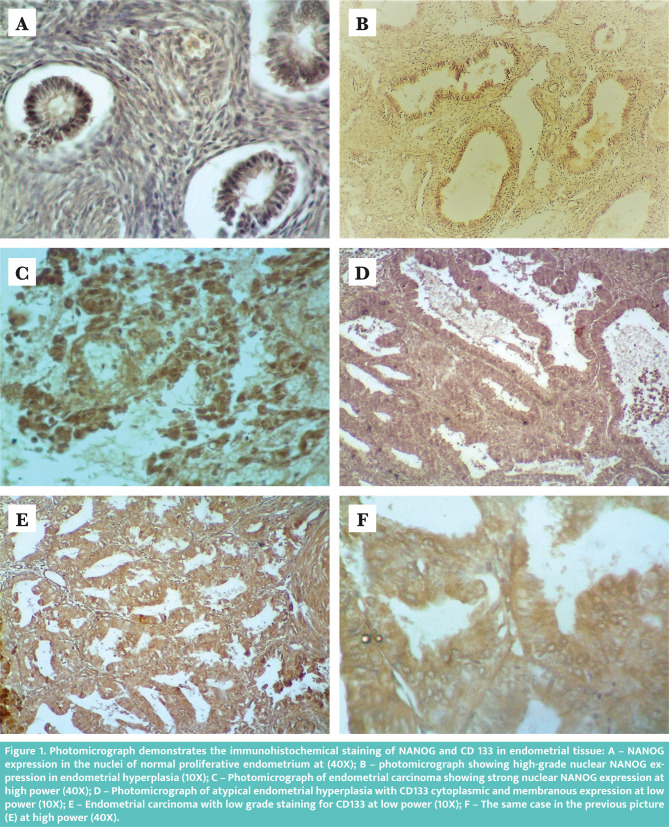
Photomicrograph demonstrates the immunohistochemical staining of NANOG and CD 133 in endometrial tissue: A – NANOG expression in the nuclei of normal proliferative endometrium at (40X); B – photomicrograph showing high-grade nuclear NANOG expression in endometrial hyperplasia (10X); C – Photomicrograph of endometrial carcinoma showing strong nuclear NANOG expression at high power (40X); D – Photomicrograph of atypical endometrial hyperplasia with CD133 cytoplasmic and membranous expression at low power (10X); E – Endometrial carcinoma with low grade staining for CD133 at low power (10X); F – The same case in the previous picture (E) at high power (40X).

NANOG expression was detected in 3 out of 20 (15%) of the normal proliferative endometrium. All of them were low expressions (as shown in Figure 1A). This expression increased in cases with hyperplasia; NANOG was positive in 18 out of 30 (60%), 8 out of 18 (44.44%) were positive in hyperplasia without atypia (6 with low expression and only 2 cases with high expression). In cases with hyperplasia with atypia 10 out of 12 were positive (2 low and 8 high), as shown in Figure 1B. NANOG expression was higher in hyperplastic conditions with atypia than those without atypia, and the difference was statistically significant (P-value 0.005). NANOG expression was detected in 38 out of 43 (88.37) endometrial carcinoma cases (as shown in Figure 1C). Table 1 summarizes the results.

**Table 1.** Comparison between positive cases of NANOG and CD 133 between the three groups (normal, hyperplastic endometrium and endometrial carcinoma.

		**NANOG**			**P value**	**CD133**			**P value**
		**Absent**	**Low**	**High**	**<0.0001**	**Absent**	**Low**	**High**	<0.0001
**Normal endometrium (n=20)**		17	3	0	19	1	0
**Hyperplastic endometrium**	**Without atypia (n=18)**	10	6	2	8	7	3
	**With atypia (n=12)**	2	2	8	2	4	6
**Endometrial cancer**		5	21	17	10	20	13

High NANOG expression was significantly correlated with high grade, deep myometrial invasion, lymph node metastasis, and high stage with p values (0.009, 0.005, 0.014, and 0.003, respectively) (Table 2).

**Table 2.** Correlation of clinicopathological parameters with NANOG and CD133 expression in 43 endometrial carcinoma cases.

**Variable**		**NANOG**			**P value**	**CD133**			**P value**
		**Absent n=5**	**Low expression n=21**	**High expression n=17**		**Absentn=10**	**Low expression n=20**	**High expression n=13**	
**Age (y)**	<55 >55	2 3	7 14	8 9	0.946	6 4	7 13	5 8	0.406
**Tumor size**	**≤4** **>4**	1 4	9 12	19 7	0.277	4 6	12 8	3 10	0.108
**Grading**	**G1** **G2** **G3**	4 1 0	14 5 2	3 6 8	0.009	6 2 2	10 5 3	2 3 8	0.058
**Depth of myometrium**	**Superficial <50%** **Deep ≥50%**	4 1	15 6	4 13	0.005	8 2	13 7	2 11	0.003
**Lymph node metastasis**	**Negative** **Positive**	4 1	7 14	4 13	0.014	8 2	14 6	2 11	0.001
**Lymphovascular** **involvement**	**Negative** **Positive**	2 3	13 8	6 11	0.241	9 1	6 14	4 9	0.003
**FIGO staging**	**I-II** **III-IV**	4 1	12 9	4 13	0.003	7 3	12 8	2 11	0.013

Regarding CD133, only one case (5%) of normal endometrium was positive for CD 133 with low expression. Normal endometrium showed less expression of CD133 than hyperplasia and endometrial carcinoma, with a highly statistically significant difference (p less than 0.0001). Hyperplastic cases with atypia express higher CD133 than those without atypia (6 out of 12 versus 3 out of 18). The staining in atypical endometrial hyperplasia is demonstrated in Figure 1D. However, this difference was not statistically significant (p-value 0.111) (Table 1). CD133 was positive in 33 out of 43 (76.74%) endometrial carcinoma cases, as shown in Figure 1E and F. It showed a significant correlation with deep myometrial invasion, positive lymph node, positive lymphovascular invasion, and high stage (p-value 0.003, 0.001, 0.003, and 0.013, respectively) (Table 2).

## Discussion

Although derived from a single clone, tumors consist of a heterogeneous cell population [13]. Recently, cancer stem cell (CSC) theory has postulated that a small subset of cells possesses some unique characteristic features like self-renewal and initiation of tumor and maintenance of its attitude with multi-lineage capacity. Some of these cells are responsible for resistance to different cancer treatment modalities like chemotherapy and radiotherapy [14]. Accumulating evidence indicates that these CSCs can be detected using a large number of markers like CD 44, CD 117, CD55, CD133, and NANOG in different tumors [15]. The present work examined the expression of two CSC markers, NANOG and CD133, in 43 endometrial carcinoma cases and compared them to 20 normal and 30 hyperplastic conditions. The expression of these 2 markers was analyzed to identify the impact of NANOG and CD133 on the behavior of endometrial tumors and their carcinogenesis.

Our study revealed that NANOG was expressed in 88.37% of endometrial carcinoma cases; this expression was higher than that in the normal and hyperplastic groups (15% and 60%, respectively). Moreover, the expression in hyperplasia with atypia is higher than those with atypia (83.33%) than hyperplasia without atypia (44.44%). The difference was statistically highly significant (p-value less than 0.0001). Interestingly, the expression of NANOG in apparently normal tissue around the tumor was higher than the normal proliferative endometrium, although it was not statistically significant. Other studies also show the high expression of NANOG in tumor samples more than in normal tissue like oral squamous cell carcinoma, salivary glands (mucoepidermoid), and glioma [5]. This result agrees with other studies demonstrating that NANOG is expressed in various precancerous lesions like laryngeal dysplasia, oral dysplasia, cervical intraepithelial neoplasia, and gastric dysplasia colonic adenoma [15]. These results suggest the possibility of using NANOG expression as a diagnostic marker to distinguish dysplasia from reactive atypia.

In our study, NANOG expression significantly correlated with bad prognostic signs like high grade, deep myometrial invasion, positive lymph node, and high stage. This implies that NANOG affects endometrial carcinoma oncogenesis, especially well-differentiated in its early stages, and their overexpression may facilitate earlier diagnosis of endometrial carcinoma. Similar results were obtained in other studies in different tumors like breast ductal carcinoma [7], colorectal carcinoma [14], and other solid tumors of the ovary, lung, kidney, esophagus, stomach, pancreas, and liver [16]. These studies have linked high expression of NANOG with poorly differentiated tumors, advanced stage, and poor survival. Furthermore, other studies correlate NANOG expression with resistance to chemo and radiotherapy. This is supported by experimental studies that show inhibition of NANOG leading to inhibition of tumor initiation, suggesting the role of NANOG in cancer development [5]. NANOG was found to be expressed in endometrial carcinoma. Transcription factors like OCT4, transcription factor 3 (TCF3), and SOX2 that regulate NANOG expression were found in endometrial CSCs and related to the potential ability for self-renewal [15]. CD 133 expressed in 76.74% of endometrial carcinoma compared to only 5% of normal tissue and 10 out of 18 in hyperplasia without atypia and 10 out of 12 in hyperplasia with atypia. Shorky *et al.* found that CD133 expression in normal and hyperplastic endometrium was nearly the same percentage as in our study [17]. High expression of CD 133 was positively correlated with bad prognostic signs like a deep myometrial invasion, positive lymph node metastasis, lymphovascular invasion, and high stage. Our study agrees with other studies which found that CD133 expression is highly associated with lymph node involvement and directly associated with tumor grading and tumor depth in gastric adenocarcinoma [18]. Also, this study agrees with another study that shows that CD133 is directly associated with tumor stage, lymph node, and distant metastasis in colorectal carcinoma [19]. Moreover, CD133 expression was correlated with bad prognostic factors like a capsular invasion, lymph node, and high stage in medullary thyroid carcinoma [20]. These results agree with Maeda *et al.*, who found that CD133 high expression in the pancreatic tumor was associated with lymph node metastasis [21].

## Conclusion

The cancer stem cell markers NANOG and CD 133 are expressed in a high percentage in endometrial carcinoma compared to normal and hyperplasia and their expression is positively correlated with the aggressive behavior of the tumor. Furthermore, high expression of these two markers was noted in apparently normal tissue around the tumor and in hyperplastic conditions with atypia, suggesting the possibility to use the expression of these markers as a diagnostic marker to distinguish dysplasia from reactive atypia. Therefore, inhibition of these markers can be a promising method to stop the progression of early cancers.

## Acknowledgments

### Conflict of interest

The authors declare no conflict of interest.

### Ethical approval

The study was approved by the Ethical Committee of Mustansiriyah University in cooperation with Al-Yarmouk teaching Hospital (approval number 150/17 from 20-10-2017).

### Consent to participate

Written informed consent was obtained from all participants in the study.

### Personal thanks

The authors would like to thank Mustansiriyah University (www.uomustansiriyah.edu.iq) Baghdad, Iraq, for support in publishing the present work.

### Authorship

MMA collected the samples and the archival material, data collection acquisition, and diagnosis of the slides. KN participated in immunohistochemistry staining. A-JAR performed the statistical analysis. All the authors contributed to study design, collection of references, slides scoring, writing the original draft and critical revision, editing of the manuscript, and technical and financial support.
